# Tumor Microenvironment of Lymphomas and Plasma Cell Neoplasms: Broad Overview and Impact on Evaluation for Immune Based Therapies

**DOI:** 10.3389/fonc.2021.719140

**Published:** 2021-12-08

**Authors:** Sudhir Perincheri

**Affiliations:** Department of Pathology, Yale School of Medicine, New Haven, CT, United States

**Keywords:** tumor microenvironment, immune based therapies, lymphomas, plasma cell neoplasms, biomarkers

## Abstract

Lymphomas and plasma cell neoplasms are a heterogenous group of malignancies derived from lymphocytes. They are a significant cause of patient morbidity and mortality. Advances in morphologic, immunophenotypic and molecular techniques have led to better understanding of the pathogenesis and diagnosis of these neoplasms. Advances in treatment, particularly immune-based therapies, increasingly allow for targeted therapies of these diseases. Mechanistic studies using animal models and clinical trials have revealed the importance of the tumor microenvironment on disease pathogenesis, progression, and response to therapy in these malignancies. Simultaneous progress in diagnostic techniques has made it feasible to generate high-resolution, high-throughput data from the tumor microenvironment with spatial context. As the armamentarium of targeted therapies and diagnostic techniques grows, there is potential to harness these advances to better stratify patients for targeted therapies, including immune-based therapies, in hematologic malignancies.

## Introduction

Lymphomas and plasma cell neoplasms are a heterogenous group of malignancies arising from lymphocytes at various stages of development. Depending on the cell of origin, morphology and immunophenotype, they are broadly categorized into non-Hodgkin lymphomas, Hodgkin lymphomas and plasma cell neoplasms (1). Non-Hodgkin lymphomas include the sub-categories of non-Hodgkin B-cell lymphomas (B-NHL) that comprise majority of cases in this group and T-cell/Natural-killer (T/NK) lymphomas. As a group, non-Hodgkin lymphomas are the seventh most common type of cancer in the United States and are expected to account for 81,560 new cases and 20,720 deaths in 2021 (2). Hodgkin lymphomas are rarer and are expected to account for 8,830 new cases and 960 deaths. For plasma cell neoplasms the corresponding numbers are 34,920 new cases and 12,410 deaths, respectively in 2021 (3, 4). The clinical course and prognosis for these heterogenous group of neoplasms is highly dependent on factors such as the specific type of disease, age, ethnicity and geographic location (1). Advances in our understanding of the molecular pathology of these diseases has resulted in considerable progress in the treatment of these diseases. Immune-based therapies using monoclonal antibodies have become part of treatment regimens for these diseases (discussed in greater detail below). More recently, greater understanding of the tumor microenvironment in these diseases has led to development of immune system modulatory agents with therapeutic potential (3, 4). Future treatment regimens are likely to rely on combinatorial strategies using these agents. These developments are going to necessitate the development of novel diagnostic and prognostic tools to facilitate optimal treatment.

## Tumor Microenvironment in Lymphomas and Plasma Cell Neoplasms

Scientific research on molecular mechanisms has expanded our understanding of cancer pathogenesis leading to advances in diagnostics and therapy. More recently, there has been increasing focus in delineating the components of the tumor microenvironment in enabling tumorigenesis and progression (5). These have led to development of several immune based therapies that have shown encouraging results both in epithelial and hematologic malignancies (6).

### Tumor Microenvironment in Lymphomas

**Figure 1:**
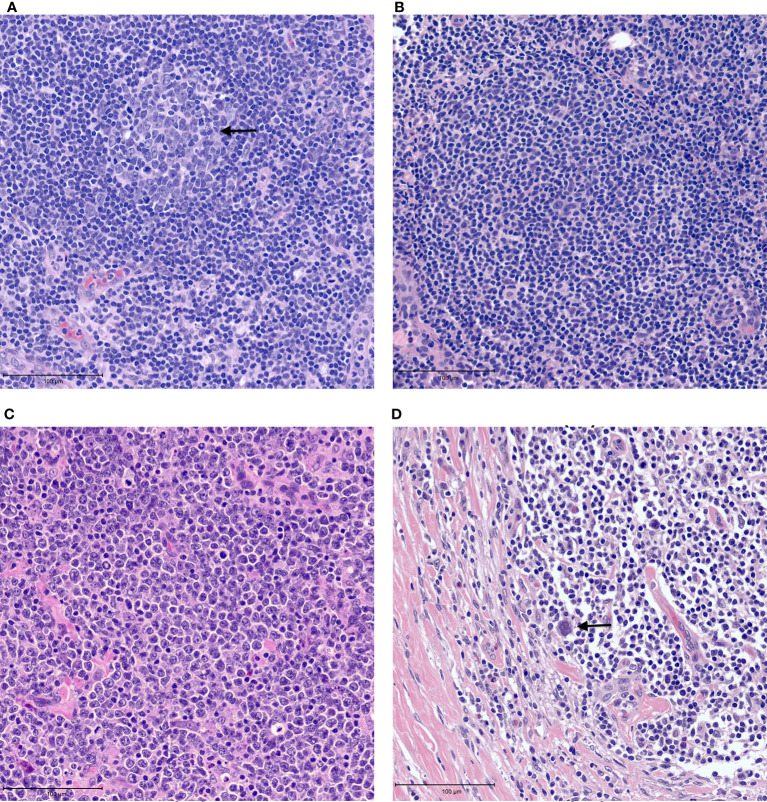
Examples of tumor microenvironment in lymphomas. **(A)** Benign lymph node with a germinal center containing tangible body macrophages (arrow). **(B)** Follicular lymphoma with neoplastic follicle. **(C)** Diffuse large B cell lymphoma showing tissue effacement by large, neoplastic cells with very few admixed small lymphocytes. **(D)** Classic Hodgkin lymphoma showing rare Reed-Sternberg cells (arrow) in a background of small lymphocytes and histiocytes with fibrosis.

The tumor heterogeneity of non-Hodgkin lymphomas is reflected in the tumor microenvironment. The histologic architecture of lymph node and extra-nodal lymphoid organs is generally comprised of B-cell predominant follicles and interfollicular T-cells (**Figure 1A**). Naive B cells exposed to antigen home to follicles where they interact with follicular dendritic cells that are antigen presenting cells. Further maturation of B-cells occurs within germinal centers where they switch-off expression of the anti-apoptotic BCL2 protein rendering them vulnerable to apoptosis. Following the process of somatic mutation and affinity maturation, B-cells either undergo apoptosis or differentiate into memory/marginal zone B-cells found in the marginal zone around the germinal centers or into long-lived plasma cells (1). Macrophages present in the germinal centers remove the apoptotic cells giving rise to the characteristic tingible body macrophages seen in benign follicles (**Figure 1A**). Morphologically, lymphomas are characterized by architectural effacement of normal lymphoid tissue. The spatial distribution of tumor cells in lymphomas is variable. Their distribution may reflect the distribution of the cell of origin as in follicular lymphomas (**Figure 1B**) (7). Follicular lymphomas are derived from follicular germinal center B-cells. Typically, these tumors arise due to an IGH-BCL2 translocation leading to BCL2 overexpression in contrast to the BCL2 switch-off seen in normal follicles (8). Spatially the follicular lymphoma cells are seen within enlarged follicles. In contrast, marginal zone lymphomas that arise from post-germinal center B-cells show an expansion of the marginal zone around the follicles. The growth of neoplastic cells in marginal zone lymphomas is often due to continuous antigenic stimulation e.g., persistent *Helicobacter pylori* infection. While eradication of the underlying infection often leads to cure, over time marginal zone lymphomas can acquire translocations that lead to cell autonomous signaling independent of the microenvironment, leading to refractory disease. In contrast to follicular and marginal zone lymphomas, the tissue architecture is diffusely effaced in diffuse large B-cell lymphomas (**Figure 1C**). The cellular composition of lymphomas is also variable. In B-NHLs, the tumor is predominantly comprised of neoplastic B-cells with fewer admixed T-cells, macrophages and stroma that comprise the tumor microenvironment. In contrast, in Classic Hodgkin lymphoma (cHL) the neoplastic Hodgkin Reed-Sternberg (HRS) cells typically form only a minor subset of the neoplastic infiltrate. The substantial cellular component of the tumor is a characteristic polymorphous population of small lymphocytes, plasma cells, histiocytes and admixed granulocytes that constitutes a morphologically unique microenvironment (**Figure 1D**).

These morphologic pattens reflect different tumor microenvironments that result from genetic aberrations in these neoplasms and the external stimuli needed for survival, proliferation, and immune response evasion. Lymphoma cells have been shown to influence their microenvironment by their ability to use homing and trafficking mechanisms to spatially colonize milieus characteristic of their non-malignant counterparts. These include expression of various adhesion molecules as well as cytokines and cytokine receptors that regulate lymphocyte trafficking between various tissue compartments (9–13). After colonization, tumor cells can shift the tissue milieu to one that promotes cell survival and growth, and immune response evasion (14–17). Cell growth results from signaling through cell surface receptors such as the B-cell receptor and Toll-like receptor as well as cytokines released from stromal cells (18–21). The evasion of immune response occurs by various mechanisms including down-regulation of major histocompatibility complex molecules, recruitment of T-regulatory cells (T-regs) at the expense of T-helper cells, and expression of programmed cell death ligands PD-L1 and PD-L2 that bind to programmed cell death protein 1 (PD-1) on CD4-positive T cells and cytotoxic T lymphocytes to induce a state of T-cell exhaustion (22–28).

Despite similar spatial localization of neoplastic cells in some lymphomas when compared to their non-malignant counterparts, there can be significant differences in their respective microenvironments. For example, the tumor microenvironment in the neoplastic follicles in follicular lymphoma has been shown to be significantly different than normal germinal centers with respect to T-cells and macrophages. Specifically, there appears to be an increase in T-regs and immune-suppressive monocytes at the expense of T-helper cells in neoplastic follicles (14, 15, 17). In cHL, the secretion of cytokines produced by and in response to HRS cells is thought to result in the characteristic polymorphous milieu that also promotes cell survival (13). Cytokines such as CCL5, CCL17 and CCL22 recruit CD4+ T-cells that constitute the major population of the tumor milieu. CSF1 and CX3CL1 recruit eosinophils and macrophages. Fibrosis, most prominently seen in the nodular sclerosis variant of cHL, is attributed to activation and proliferation of fibroblasts by IL-13, TNF-α and fibroblast growth factors by HRS cells (13). Interaction between membrane-bound or secreted ligands from the microenvironment and cell surface receptors on HRS cells results in activation of canonical signaling pathways such as the JAK-STAT, NF-kB and BCR pathways. These ligand-based mechanisms are complimented by gene mutations that cause constitutional activation of these signaling pathways (29). In highly aggressive tumors such as Burkitt lymphoma, the tumor cells often acquire mutations that result in cell autonomous growth signals (30). Most cases of Burkitt lymphoma harbor translocations of the MYC gene, typically juxtaposing it with enhancer genes of the IGH locus, and less frequently the IGK and IGL loci resulting in upregulation of c-myc, its target genes and target microRNAs. These and other mutations result in cell-autonomous growth signaling. These tumors are, therefore, less likely to need stromal signals for survival resulting in a characteristically sparse tumor microenvironment (31–34).

A better understating of expression, distribution, and interaction of PD-1 and PD-L1 in the tumor microenvironment has been critical in the development of immune checkpoint blockade therapy (CBT) in cancer therapy. CBT involves blockade of the PD-1/PD-L1 axis to enhance the therapeutic effects of anti-tumor immune response (35). PD-1 is expressed predominantly on activated T cells, natural killer cells, dendritic cells, macrophages, and B-cell subsets. PD-L1 is expressed on a wide variety of hematopoietic and non-hematopoietic cells including stromal cells and can also be expressed by tumor cells. In the context of lymphomas, PD-1 expression is seen in tumor infiltrating lymphocytes, as well as the neoplastic cells in some types of B- and T-cell lymphomas (36). PD-L1 expression is seen in a wide variety of neoplastic cells in various lymphomas including cHL, Primary mediastinal large B cell lymphoma, and extra-nodal NK-T cell lymphomas among others (37). PD-L1 expression is also seen in some immune cells within the tumor microenvironment including tumor infiltrating lymphocytes as well as tumor associated macrophages (TAMs).

### Tumor Microenvironment in Plasma Cell Neoplasms

Unlike lymphomas that often present in nodal tissues, plasma cell neoplasms are primarily diseases of the bone marrow. The marrow stromal niche in plasma cell neoplasm is occupied by a variety of cells including T-regs, NK cells, macrophages, dendritic cells, bone marrow stromal cells, endothelial cells, osteoblasts, and osteoclasts embedded in an extracellular matrix (38–40). The stromal niche has been shown to influence homing and adhesion of plasma cells by expression of adhesion molecules and cytokines (41–43). It also appears to aid immune evasion by mechanisms similar to those seen in lymphomas. For example, increased T-regs have been reported in the peripheral blood of neoplasms patients with plasma cell neoplasms (44). Interaction between myeloma cells and T-regs results T-reg expansion by a type 1IFN-dependent positive feedback loop (45). In response to stromal signals, plasma cell neoplasms cells express PD-L1 that interacts with PD-1 protein in T-cells, leading to immune exhaustion (46, 47). A caveat to these observations is that they are based on qRT-PCR and flow cytometry studies on small sample sets that include both patient samples and cell lines; larger studies are needed to validate these observations.

## Immune-Based Therapy in Lymphomas and Plasma Cell Neoplasms

Current therapy for non-Hodgkin lymphomas is dependent on various factors including the pathologic subtype of lymphoma, stage at presentation, and performance status of the patients. The treatment can range from watchful waiting to combination modality regimens followed by stem cell transplant. Salvage regimens are used to treat relapsed disease (48). Therapy for Hodgkin lymphoma is also influenced by stage and risk factors and can range from chemotherapy alone to combination modality treatment, and salvage therapy for progressive disease (49). Treatment for symptomatic myeloma typically involves immunomodulatory drugs and proteasome inhibitors (50, 51). For all three entities, immune based therapies are increasingly part of the therapeutic armamentarium in treatment regimens (3, 40).

### Immune-Based Therapy in Lymphomas

Immune based therapies have been used for treatment of lymphomas for more than two decades (3). The earliest success was with rituximab, a chimeric monoclonal antibody targeting the B-cell marker CD20 (52–55). Since then, several other antibodies targeting B- and T-cell markers including CD30, CD19 and CCCR4 have been evaluated in lymphoma treatment with many such drugs receiving regulatory approval (56–59). Initial targeted monoclonal antibodies used complement dependent cytotoxicity or antibody-dependent cytotoxicity for anti-tumor effect. More recently, attempts have been made to increase their efficacy by conjugating them to cytotoxic drugs (60, 61). Bispecific T cell engager antibodies (BiTEs) that are designed to target both tumor antigens and T-cells to bring the tumor cells in close physical proximity to the T cells for enhanced anti-tumoral effects are also being evaluated (62, 63). There is mechanistic evidence that the tumor microenvironment can impact response to therapy. In cell-culture based studies CXCR-4 dependent interaction of lymphoma cells with stromal cells has been shown to protect lymphoma cells from anti-CD20 monoclonal antibody induced apoptosis (64). Studies have also shown induction of microRNAs impacting levels of proapoptotic proteins and upregulation of cell survival signals (65). As in solid organ epithelial tumors, there is intense interest in harnessing the power of CBT in lymphoid malignancies. Downstream signaling from the PD-1 receptor in T cells due to PDL-1 overexpression in tumor cells leads to immune exhaustion and helps tumors evade immune response. Blockade of this signaling pathway with monoclonal antibodies targeting either the PD-1 receptor or its ligand is predicted to enhance antitumor immune response (35). The results of immune checkpoint blockade in lymphomas have been mixed. The strongest response has been seen in lymphomas associated with high PDL-1 expression including Hodgkin lymphoma, primary mediastinal large B cell lymphoma and EBV-associated lymphoproliferative disorders (66–71). In contrast, the efficacy of CBT in other lymphomas such as DLBCL and chronic lymphocytic leukemia (CLL) have been less impressive (72, 73). Other strategies targeting the host immune response to lymphoma are being evaluated e.g., antibodies to the CD47 molecule that suppresses macrophage-induced phagocytosis by binding to signal regulatory protein-alpha (74).

The data with engineered adoptive cell therapies is promising in lymphoma therapy. Details of chimeric antigen receptor T cells (CAR-T) based therapy have been reviewed elsewhere (75). Briefly, CAR-Ts are created by transducing genetic material into patient’s own T-cells using lentiviral or retroviral vectors. The chimeric receptor contains an antigen binding extracellular domain that targets a tumor antigen coupled to an intracellular signaling domain, including chimeric domains derived from costimulatory proteins. This design allows the CAR-T cells to respond to tumor antigen without MHC presentation. The CAR-T cells are infused after lymphodepletion chemotherapy. Upon antigen recognition, the receptors cluster together to trigger a T-cell activating signaling cascade. CAR-T therapy targeting CD19 has been approved for the treatment of relapsed refractory aggressive B-NHLs and acute lymphoblastic leukemia following impressive and sustained response in clinical trials (76–78). Prospective clinical trials to evaluate expanded use of CAR-T as an alternative to stem cell transplant and in other B-NHLs are ongoing including combinatorial strategies with CBT and immunomodulatory therapy (3). Lack of long-term response due to emergence of target antigen negative cells (also known as antigen escape) has led to combinatorial antigen targeting including CD19 and CD20 or CD19 and CD22 (79–81). In contrast to CBT, CAR-T responses in Hodgkin lymphoma and T-cell lymphomas have not been impressive (82, 83).

Other immune based therapeutics in lymphoma include immunomodulators and small molecule inhibitors such as lenalidomide and ibrutinib. Lenalidomide and other thalidomide analogs exhibit immune modulatory effects by altering cytokine production, regulating T cell co-stimulation and augmenting NK cell cytotoxicity (84, 85). Ibrutinib and acalabrutinib are small molecule inhibitors that inhibit Bruton tyrosine kinase (BTK) that is part of the B cell receptor signaling pathway (86–89). In addition, ibrutinib can modulate immune response by increasing Th1 T cell response at the expense of Th2 T cell response by blocking IL-2 inducible kinase (90). Finally, observations in non-Hodgkin lymphomas have shown that treatment with these kinase inhibitors leads to the mobilization of lymphoma cells from their stromal niches into the bloodstream which may be an added component of their efficacy (91).

### Immune-Based Therapy in Plasma Cell Neoplasms

Several immunotherapy approaches are being used in treatment of plasma cell neoplasms, both as part of standard-of-care treatments and in clinical trials (92). Lenalidomide is used in first line treatment of both standard-risk and high-risk plasma cell neoplasms (51). Targeted antibodies are now FDA-approved for treatment of plasma cell neoplasms. Anti-CD38 antibodies (daratumumab) in combination with immunomodulators has shown improved outcomes in relapsed refractory plasma cell neoplasms. The mechanism of action includes complement- and antibody-mediated cytotoxicity, and suppression of T-regs and regulatory myeloid populations (93). In contrast to B-NHLs results of CBT therapy in plasma cell neoplasms have been less impressive (94, 95). Combination immunotherapy approaches after autologous stem cell transplant are being evaluated based on encouraging data from preclinical trials (96). Immune modulatory drugs are now routinely used in treatment of symptomatic myeloma in combination regimens where they induce plasma cell apoptosis in addition to immune stimulatory effects. Other immunomodulatory regimens are being evaluated in early clinical trials. CAR-T therapy targeting B cell maturation antigen (BCMA) has shown promise (97, 98). BCMA is expressed primarily in plasmablasts and plasma cells in the bone marrow with no detectable expression in naïve B cells and hematopoietic cells (99, 100). The BCMA expression levels are much higher in neoplastic plasma cells compared to normal plasma cells. In response to signaling from its ligands APRIL and BAFF, BCMA signaling leads to activation of pro-survival pathways (38). Despite impressive early response in trials, persistent and durable response has not been seen, likely due to antigen escape and immunosuppressive effect of the bone marrow tumor microenvironment (101). Other immunomodulatory techniques targeting BCMA including targeted antibodies, BiTE antibodies, and CAR-T therapies to other antigens such as CD138 are under clinical development (102, 103).

## Impact on Diagnostics, Prognostics, and Biomarkers

The diagnostic work-up of lymphomas and plasma cell neoplasms incorporates morphologic evaluation, immunophenotyping by tissue immunohistochemistry and multiparametric flow cytometry, and ancillary studies. The ancillary studies include cytogenetics, fluorescence *in situ* hybridization (FISH), molecular studies such as PCR for immunoglobulin heavy chain rearrangement, and targeted mutation detection for specific disease entities (104). While substantial information about disease behavior and prognosis can be obtained by these studies, they may provide only limited information regarding the tumor microenvironment and the potential for response to immune based therapies. There is a need to broaden the diagnostic and prognostic modalities to better predict disease response to immune-based therapies in lymphomas and plasma cell neoplasms. Some specific diagnostic modalities and their potential applications are briefly discussed below. While some techniques can be applied routinely in clinical diagnostic labs at the current time, other techniques exist currently more in the realm of clinical research that with time will probably supplant the current techniques.

### Immunophenotyping

Immunohistochemistry (IHC) which involves interrogation of formalin-fixed paraffin embedded (FFPE) tissue with specific antibodies for specific antigens, and multiparametric flow cytometry are indispensable tools in clinical hematopathology diagnostic labs (104). These methods have potential utilities in assessing tissue for response to immune-based therapy. In solid organ tumors involving the lung and genitourinary tract for example, an FDA-approved immunohistochemistry based diagnostic assay for PD-L1 is used to predict response to CBT (105, 106). There are currently no FDA-approved IHC diagnostic assays to identify patients that will respond to CBT in hematologic malignancies. Analysis of PD-1 and PD-L1 expression patterns in hematologic malignancies show promising albeit sometimes contradictory results. For example, high proportion of PD-L1 positive macrophages or PD-1-positive T-cells is associated with favorable outcomes in primary testicular lymphoma (107). Similarly in a cohort of *de novo* DLBCL, increased myeloid derived PD-L1 cells correlated with STAT3 and macrophage gene expression, and improved outcomes in a subset of patients (108). A study utilizing 3-marker fluorescent multiplex immunohistochemistry coupled with automated immunofluorescent analysis concluded that the PD-1/PD-L1 expression and interaction is associated with adverse prognosis in DLBCLs with significant T cell infiltration (109). The variability of results in some of these studies may be attributable to the different antibody clones and experimental methods used in these studies illustrating the need for standardization of these assays across clinical labs prior to widespread diagnostic use. The fact that many of the antigens targeted by immune-based therapies such as CD20, CD19 and CD30 are evaluated during routine lymphoma diagnostic workup is fortuitous. However, the utility of IHC assays to predict disease response to CBT and targeted immunotherapy in hematologic neoplasms is limited in the absence of prospective studies. While it is logical to assume that immunopositivity and the level of antigen expression as assessed by immunophenotyping will correlate with disease response, one should be cautious with such assumptions. Individual studies looking at the correlation of antigen expression by IHC and response to targeted therapy have sometimes shown variable correlation as in the case of CD30 (110, 111). Additionally, lab-to-lab variability in the individual clones of antibody used and in staining protocols can lead to subjective interpretation and poor reproducibility of IHC and flow cytometry studies (112–114). On the other hand, immunomodulatory techniques themselves have potential to impact diagnostic assessment by these techniques. For example, CD20 staining can yield negative IHC results after treatment with rituximab due to antigen masking and down-regulation of CD20 expression (115). Bright CD38 expression is often used in assessing plasma cells by flow cytometry; treatment by daratumumab can lead to interference and artifactual results with laboratory assays for monitoring disease (116, 117). As the arsenal of targeted antibodies grows, diagnosticians need to be keenly aware of such iatrogenic artifacts during diagnostic and prognostic work up. Documentation of increased macrophages, cytotoxic T cells and NK cells by IHC has been shown to correlate with outcomes in cHL (118–121). Current immunohistochemical workup in clinical labs typically uses one antibody on one slice of tissue at a time on glass slides. The development of high-throughput, multiplex immunohistochemical methods opens the possibility of simultaneous evaluation of multiple markers on a single slice of tissue. The individual markers can be evaluated for their expression status and spatial distribution facilitating assessment of the tumor microenvironment by immunophenotypic methods (122–124). Immunophenotypic evaluation may also have utility in predicting response in CAR-T therapy. For example, a population of CD27^+^PD-1^-^CD8^+^ cells expressing high levels of the IL-6 receptor has been shown to correlate with therapeutic response (125).

### Digital and Computational Pathology

IHC assays performed on FFPE tissue are easy to adopt in anatomic pathologic labs. However, interlaboratory variability in protocols and subjective variation in manual interpretation can lead to poor reproducibility of assays with impact on treatment and clinical course. The evolution of digital pathology where whole slide imaging scanners are used to digitize glass slides and render images in digital formats has aided the development of automated, reproducible, computer aided diagnostic tools that promise to be the next frontier in tissue-based diagnostics (126). WSI technology builds images of whole slides by stitching together multiple images of tissue sections on slides (127). WSI scanners have now been approved by the FDA for purposes of rendering anatomic pathology diagnoses (128). The College of American Pathology has published guidelines for validation and adoption of digital pathology techniques in clinical settings (129). Digital analyses tools including machine learning algorithms can be applied on digitized histology images for reproducible and quantitative assessment of tumors and their microenvironment. Multiplex approaches can yield high-complexity data with regard to spatial expression of multiple markers in the tumor microenvironment (130). In cHL for example, multiplex IF has shown the association of tumor microenvironment with CTLA-4-positive T cells that are PD-1 negative (131). Data shows that multiplex methods may be better at predicting response to CBT than standard IHC or gene expression profiling methods (132). These analyses can be extended to assess response to immunotherapy by assessing for distribution of components of the immune system such as regulatory T cells and macrophages. A limiting factor in the development and application of machine learning algorithms in computer aided diagnosis is the need for large, high quality data sets to train these algorithms (133). The performance metrics and portability of these algorithms across datasets can also be impacted by pre-analytic variables such as slide scan quality, need for additional image processing and input from human pathologists for accurate interpretation (134, 135). While multiplex IF provides high-resolution data about the tumor microenvironment, complexity of analyses, cost and time considerations currently limit the applicability of this technique in clinical settings. For practical purposes, gene expression analysis is currently utilized to assess characteristic signatures in diagnostic settings.

### Molecular Techniques

Ancillary molecular techniques are a significant part of current diagnostic workflow in lymphoid and plasma cell malignancies (104). The presence of specific cytogenetic abnormalities, specific gene mutations, and other molecular findings such as IGH hypermutation status impact disease prognosis in various lymphomas and plasma cell neoplasms, and factor into the calculation of risk stratification scores of individual entities (1). These studies reveal little about the effect of tumor microenvironment on disease course since the spatial context is often lost with these techniques. However, studies have shown the potential utility of gene expression profiles that appear to reflect the tumor microenvironment in predicting response to immune based therapies. For example, in DLBCL gene expression profiles derived from non-malignant cells have shown association with response to R-CHOP therapy. A signature enriched for genes associated with components of the extracellular matrix deposition and histiocytic infiltration was associated with good behavior whereas a signature associated with angiogenesis was associated with poor prognosis (136). Similarly in follicular lymphoma, gene expression profiles associated with macrophages are correlated with different prognoses. Expression of a set of genes enriched for T-cell markers and genes highly expressed in macrophages was associated with better prognosis while a signature enriched for genes highly expressed in dendritic cells, macrophages or both was associated with worse prognosis (137). The refinement of techniques that allow for spatial single cell sequencing from FFPE tissue with the potential to deliver high-resolution molecular data of the tumor microenvironment with spatial context are particularly exciting developments in this realm (138). Techniques such as CODEX (for CO-Detection by indEXing) that utilizes DNA barcodes, fluorescent dNTP analogs and an *in-situ* polymerization based indexing procedure to iteratively detect antibody binding events have recently been described (139). This methodology allows for single cell antigen quantification in tissue sections, and unlimited levels of multiplexing to map cell types in tissues. The continued development of multiplexed and high-dimensional imaging methods, and their application in translational research are likely to lead to a better understanding of the tumor microenvironment in malignancies and their impact on response to therapy (140).

The power of using a combinatorial approach to dissect tumor microenvironment in lymphoid malignancies is demonstrated by a recent study that used FISH, chromogenic IHC, and multiplex immunofluorescence microscopy with cell phenotyping followed by spatial analyses of the cell phenotypic data to characterize the PD1/PD-L1 pathway in the tumor microenvironment of a multi-institutional cohort of T-cell/histiocyte-rich large B-cell lymphomas (THRLBCL) (141). The authors found frequent PD-L1/PD-L2 copy gain or amplification in the large malignant B-cells of THRLBCL. Using sophisticated spatial image analyses to characterize the distribution of immune cells and their PD1/PDL1 expression status, the authors were able to develop spatially resolved immune signatures that distinguish TCRLBCL from cHL and DLBCL.

## Conclusion

Recent advances in our understanding of the tumor microenvironment have led to better understanding of pathogenesis of lymphomas and plasma cell neoplasms. Concurrent advances in immune based therapies have highlighted the importance on the tumor microenvironment on disease course and response to therapy. Advances in diagnostic modalities are likely to lead to better biomarker identification, patient risk stratification and theranostic prediction in hematologic malignancies.

## Author Contributions

The author researched the topic and wrote the manuscript.

## Funding

Salary support for the author was provided by the Department of Pathology, Yale School of Medicine, Yale University.

## Conflict of Interest

The author declares that the research was conducted in the absence of any commercial or financial relationships that could be construed as a potential conflict of interest.

## Publisher’s Note

All claims expressed in this article are solely those of the authors and do not necessarily represent those of their affiliated organizations, or those of the publisher, the editors and the reviewers. Any product that may be evaluated in this article, or claim that may be made by its manufacturer, is not guaranteed or endorsed by the publisher.
